# The Hospitals and Women Students

**Published:** 1916-07-22

**Authors:** W. D. Halliburton

**Affiliations:** King's College, London, Strand, W.C.


### THE EDITOR'S LETTER-BOX.

### The Hospitals and Women Students.

*To the Editor of* The Hospital.

Sir,—In the annotation on this subject in your issue of to-day I notice an error. You state that King's College Hospital is among those who have opened their schools to women. This is not the case. King's College, London, has taken this step, but King's College Hospital, which is now a separate school (united to King's College only by name and sentimental ties), has not yet seen fit to follow suit.—Faithfully yours,

W. D. Halliburton.

King's College, London,
Strand, W.C., July 15, 1916.

